# Dyspepsia

**Published:** 1870-01

**Authors:** 


### HALL'S
### JOURNAL OF HEALTH.

VOL. XVII.] JANUARY, 1870. [NO. 1.

"HEALTH IS A DUTY."—ANON.

"Men consume too much food and too little pure air;
They take too much medicine and too little exercise."—ED.

"I labor for the good time coming, when sickness and disease, except congenital, or from accident, will be regarded as the result of ignorance or animalism, and will degrade the individual, in the estimation of the good, as much as drunkenness now does."—IBID.

Our legitimate scope is amost boundless for whatever begets pleasurable and harmless feelings, promotes Health; and whatever induces disagreeable sensations, engenders disease.

We aim to show how disease may be avoided, and that it is best, when sickness comes, to take no medicine without consulting a physician.

### DYSPEPSIA.

Chronic dyspepsia, that which lasts for weeks and months and weary years, to make a whole lifetime a misery, is always caused by habitually eating more than the stomach can digest, and is never cured by medicine, nor ever can be, because nothing can digest food healthfully, so as to impart nutriment and strength to the system, but gastric juice, called "gastric," because that is the Greek name for the stomach.

This gastric juice is a fluid which accumulates gradually in certain vessels which are found along the sides of the stomach, and when they get full a sense of hunger is felt, which will not be appeased until these vessels are emptied; they are made to empty themselves by the food taken into the stomach, and touching them; just as sometimes the reader has experienced when being pretty hungry, and a mouthful of food being taken, the feeling of a gush of liquid from the sides of the cheeks.

GASTRIC JUICE.

This gastric juice is sent to these vessels on the sides of the stomach by a power wholly beyond our control, and in a manner inexplicable to us; we can only take cognizance of the result. What we know certainly is simply this: that the amount of gastric juice is proportioned to the wants of the system. The body is always wearing out like any sewing-machine; and like a machine, it constantly needs repair; the body is made to wear out by its motion; there can be no motion without waste of substance, because all motion of machinery and of body implies friction, and friction causes waste. The more motion, the more waste; the more waste, the more want of repair; the more want of repair, the more rapidly and the more in quantity is the gastric juice sent to the stomach, as a kind of messenger from the outposts to give information that supplies are needed. The stomach, so to speak, has learned by experience that taking food into it meets the wants of these messengers, and this in time becomes an ineradicable impression. We must carry out the metaphor in order to make an important practical impression on the mind of the general reader so as to prepare the way for convincing him that there is only one way to cure dyspepsia radically and permanently. The messengers have so often gone away satisfied when a full meal has been taken, that the stomach concludes that to satisfy the messengers for help, it is only necessary to have a full meal. But once upon a time the messengers came, and made their demands for new supplies; the stomach answered by calling for a good dinner, which was given as usual; but the messengers were not satisfied; they still pressed their demands, and the stomach called for more food; and more food was eaten, with the same result, that the person still remained hungry; always eating, always hungry, never satisfied; this is one of the most common symptoms of dyspepsia. Just at this point, we meet the almost universal obstacle in the cure of dyspepsia; both nature and the patient make a fatal mistake—fatal as far as a prompt and permanent cure is concerned. Blind instinct makes her want known to the stomach—a want of supplies to take the place of the wastes of the system, so as to keep up its strength and health; but the stomach calls for food, as food seemed to have met these demands formerly; the messengers, however, wanted nourishment, not food; it wanted the essence of the food to be sent to the outposts, but the stomach had been overworked; it was not able to extract that essence, only the semblance of it, which, not being the real material failed to satisfy the system's wants; the stomach is hungry, or excites hunger, and the man eats, and eats, and eats; so urgent is his appetite sometimes, that the dyspeptic feels, about an hour before the regular meal, as if he would almost die if he had to wait an hour; and many times, if he waits that hour, he has little or no appetite, showing that the desire for food awhile ago was fictitious; but the digestive organs having a little time longer to work on the material already in them, extracted some more nutriment, and sent it on to the outposts, and thus pacified them, as craving creditors are sometimes pacified, at least for the time, by an infinitesimal dividend of a failing debtor, or company, or bank.

### CURE OF DYSPEPSIA.

To be sure this is not physiologically and critically exact, but it is largely so, and enables us to communicate more intelligibly an important practical fact, which, if critically examined in all its bearings, suggests the steps necessary to be observed in the cure of dyspepsia.

The first is that the patient should wait for the regular times of eating, however ravenously hungry he may feel, and he has, to help him, the encouraging fact that, if thus treated, this ravenous appetite decreases every day, and within a week he can wait for his regular meal without special discomfort.

It is fair to infer from what has been said, that a dyspeptic would hasten his cure if he ate but twice a day, for two reasons: the stomach would have a longer time of rest, and would inevitably have more power to work on what might be introduced into it.

The inference is quite as legitimate, that the longer the time allowed the digestive functions to work on the food eaten, the more nourishment would be derived, the more perfectly that food would be worked up.

SIGN OF DYSPEPSIA.

If a man is hungry within an hour, more or less, after a regular meal, he is a dyspeptic beyond question, and it shows that the stomach is not able to work up what he has eaten so as to get nourishment out of it; but to eat again, and thus impose more work when it could do nothing for what had been already eaten, is an absurdity; and yet all dyspeptics who eat whenever they are hungry, do this very thing, and thus aggravate and protract their sufferings. It is precisely as when a coal fire is kindling, the more coal is put on the more certainly the fire is put out, because coal will only kindle by enough heat being concentrated on it to warm it up thoroughly; but if there was not heat enough in the kindling to saturate what coal there was already around it, the addition of more coal only increases the distribution of heat on a larger mass of it, when the object should be to concentrate what heat there was on a smaller amount of coal. So in dyspepsia we must concentrate the feeble efforts of the weak stomach on a smaller amount of food, and then the door will open to a happy cure.

It was before stated that by some mysterious agency the waste and wear of the system had the effect to accumulate gastric juice on the walls of the stomach, and that its presence there excited the sensation of hunger, as the collection of urine in the bladder excites a desire for its discharge.

EXERCISE.

But if waste and wear excite hunger in the end, and exercise causes this waste and wear, then the dyspeptic must exercise, so as to cause this waste and wear, which in turn creates the gastric juice which causes digestion. The steps are these: Nutriment is wanting in dyspeptics to supply the out posts of the system with materials for repair; this nutriment is extracted from the food we eat by a process which we call digestion; this process is accomplished by means of the gastric juice in the stomach, which dissolves the food from without inward, as small bits of ice in a glass of water are dissolved from without inward, layer by layer, until the ice has disappeared, and all is water.

But this gastric juice is in a sense the result of the wear and waste of the system caused by exercise, hence the argument has all the logicality of a demonstration of Euclid. Dyspepsia is cured by muscular exercise, voluntary or involuntary, and cannot be cured in any other way, because nothing can create or collect gastric juice, except exercise; it is a product of the human machine—Nature only can make it.

There is a most beautiful truth in this connection, as showing the economy of means to ends in the great Architect of our material frames. All know that the wastes of machinery, and the oils used for lubricating purposes, must be constantly cleaned away by the watchful engineer. The watch becomes clogged up by these wastes in time, and unless cleaned it would stop forever. But great Nature removes her own wastes by such an arrangement within us, that the motion of every muscle tends to push out of the system all that does not belong to it—all that is in it which does not subserve the purposes of health; so that the more exercise we take, the more perfectly are the waste matters of the system worked out of it. No wonder then that those who are a great deal in the open air, those who work, keep the human machine in healthful action, are vigorous in body, and have a litheness of motion, which add largely to the efficiency of their labor; no wonder then that persons who are indoors a great deal, take but little exercise, and sit about the house almost without an end or aim in life, soon have their systems clogged up, and are dull and sad and stupid and ailing; or if the clogging up affects the body in another direction, they are fretful, peevish and spiteful, and revel in ill-natured gossip, in misconstruing motives, and in sowing the seeds of family disagreements and neighborhood alienations.

The point to which the reader's admiration is directed is the

WONDERFUL ECONOMY

of nature, by which the very exercise necessary for working the wastes of the body outside of itself, prepares an abundant supply of gastric juice, which first causes hunger, and then so acts upon the food eaten as to secure from it the extraction of the nutriment which is to repair the waste and wear of the body, builds anew the human fabric, and keeps it running without a stoppage, for a

HUNDRED YEARS!

For a more detailed explanation of these principles, and their application to the cure of biliousness, neuralgia, etc., see "Health by Good Living," sent from this office, post-paid, for one dollar and seventy cents.

